# Target deletion of complement component 9 attenuates antibody-mediated hemolysis and lipopolysaccharide (LPS)-induced acute shock in mice

**DOI:** 10.1038/srep30239

**Published:** 2016-07-22

**Authors:** Xiaoyan Fu, Jiyu Ju, Zhijuan Lin, Weiling Xiao, Xiaofang Li, Baoxiang Zhuang, Tingting Zhang, Xiaojun Ma, Xiangyu Li, Chao Ma, Weiliang Su, Yuqi Wang, Xuebin Qin, Shujuan Liang

**Affiliations:** 1 Key Lab for Immunology in Universities of Shandong Province, School of Clinical Medicine, Weifang Medical University, Weifang, 261053, P.R.China; 2 Department of Neuroscience, Temple University School of Medicine, Philadelphia, PA19140, USA

## Abstract

Terminal complement membrane attack complex (MAC) formation is induced initially by C5b, followed by the sequential condensation of the C6, C7, C8. Polymerization of C9 to the C5b-8 complex forms the C5b-9 (or MAC). The C5b-9 forms lytic or non lytic pores in the cell membrane destroys membrane integrity. The biological functionalities of MAC has been previously investigated by using either the mice deficient in C5 and C6, or MAC’s regulator CD59. However, there is no available C9 deficient mice (*mC9*^−/−^) for directly dissecting the role of C5b-9 in the pathogenesis of human diseases. Further, since C5b-7 and C5b-8 complexes form non lytic pore, it may also plays biological functionality. To better understand the role of terminal complement cascades, here we report a successful generation of *mC9*^−/−^. We demonstrated that lack of C9 attenuates anti-erythrocyte antibody-mediated hemolysis or LPS-induced acute shock. Further, the rescuing effect on the acute shock correlates with the less release of IL-1β in *mC9*^−/−^, which is associated with suppression of MAC-mediated inflammasome activation in *mC9*^−/−^. Taken together, these results not only confirm the critical role of C5b-9 in complement-mediated hemolysis and but also highlight the critical role of C5b-9 in inflammasome activation.

The complement system, an important component of innate and acquired immunity, has the capacity to lyse and thereby inactivate pathogenic microorganisms^1^. However, this response also contributes to the pathogenesis of many chronic immune and inflammatory diseases^1^,^2^,^3^,^4^. The complement system consists of approximately 30 soluble and membrane-bound proteins and is activated by 3 distinct pathways—the classical, lectin and alternative cascades—either on the pathogen surface or in the plasma. All three activation pathways converge at the C3 level, leading to the subsequent formation of C5 convertase. C5 convertase then cleaves C5, thus forming C5b and C5a. The terminal complement activation pathway is induced initially by C5b, and this is followed by the sequential condensation of C6 to C5b6 and then C7, C8, and C9. The polymerization of C9 bound to the C5b-8 complex forms C5b-9, or the membrane attack complex (MAC), an end product of the complement activation pathway. The MAC forms a lytic pore in the lipid bilayer of the membrane, which allows for the free passage of solutes and water across the membrane and destroys membrane integrity, thus leading to the destruction of foreign pathogens and the death of infected cells^1^,^2^,^3^,^4^. To protect host cells from MAC attack, more than ten plasma- and membrane-bound inhibitory proteins have evolved that restrict complement activation at different stages of the complement pathway^1^,^2^,^3^,^4^. Moreover, three membrane proteins expressed on the surface of the majority of host cell types inhibit autologous complement activation, thereby protecting cells from complement-mediated injury^5^. These regulators include CD55, CD46, and CD59. CD55 inactivates C3 (C4b2a and C3bBb) and C5 (C4b2a3b and C3bBb3b) convertases by accelerating the decay of these enzymes^6^. CD46 acts as a cofactor for the cleavage of cell-bound C4b and C3b by the serum protease factor^7^. CD59 is the most important membrane inhibitor, which restricts C5b-9 formation by preventing C9 incorporation and polymerization^1^,^2^,^3^,^4^.

Over the past 15 years, the biological functionalities of the terminal complement pathway, including the components C5b-7, C5b-8 and C5b-9 (MAC), have previously been investigated *in vitro* in cell culture systems or *in vivo* in mice deficient in the crucial MAC regulator CD59^8^,^9^,^10^ (leading to increased MAC formation) or mice deficient in C5^11^ or C6^12^ (leading to decreased MAC formation). Although the MAC forms a lytic pore, leading to rapid destruction of cells, most nucleated cell targets resist lysis through a combination of ion pumps, membrane regulators and active recovery processes^13^. Formation of the MAC at a sublytic concentration in a cell membrane *in vivo* activates signaling cascades^14^ and releases inflammatory mediators, thus leading to the activation of nucleated cells and mediating cellular processes such as proliferation, inflammation, and thrombosis^15^,^16^. Although this process has been recognized for thirty years, there is no consensus signaling pathway for sublytic concentrations of the MAC^13^. Recent experimental evidence has indicated that 1) complement activation by cholesterol crystals promotes lipopolysaccharide (LPS)-mediated caspase-1 activation *in vitro*^17^,^18^, and 2) caspase-1 activation links sublytic MAC-mediated inflammation to the induction of interleukin (IL)-1β maturation and its release *in vitro* and *in vivo*^17^. Specifically, the study by Laudisi, *et al*. using *C6*^−/−^ mice, which show impaired terminal complement cascade activation, has demonstrated that the terminal complement cascade activation is indispensable for NLRP3 inflammasome-mediated IL-1β maturation^17^. However, the contribution of C5b-9-induced IL-1β to the pathogenesis of human diseases, as modeled in animal systems, remains to be investigated. In addition, it has been recognized for over thirty years that the C5b-7 and C5b-8 complexes are also able to form a nonlytic pore, thereby mediating a hemolytic effect, killing bacteria and triggering the activation of signaling pathways *in vitro*, although their biological function and relevance remain to be determined *in vivo*^19^,^20^,^21^. Further, emerging evidences suggest that the bio-products of complement activation produced in complement activation has distinct roles in pathological^22^,^23^ and physiological conditions^24^.

To elaborate on these previous discoveries and to better understand the role of C5b-9, a terminal complement activation product, we herein report the successful generation of C9 knockout (*mC9*^−/−^) mice through a targeted deletion approach. We also demonstrate that the loss of C9, which leads to decreased C5b-9 production, attenuates anti-erythrocyte antibody-mediated hemolysis and LPS-induced acute shock. Furthermore, the rescuing effect on the acute shock correlates with the less release of IL-1β in *mC9*^−/−^, which is associated with suppression of MAC-mediated inflammasome activation in *mC9*^−/−^ mice. Together, these results not only confirm the critical role of C5b-9 in complement-mediated hemolysis but also highlight the critical role of C5b-9-induced inflammasome activation in LPS-induced shock.

## Results

### Generation of *mC9* ^−/−^ mice

**Figure 1:**
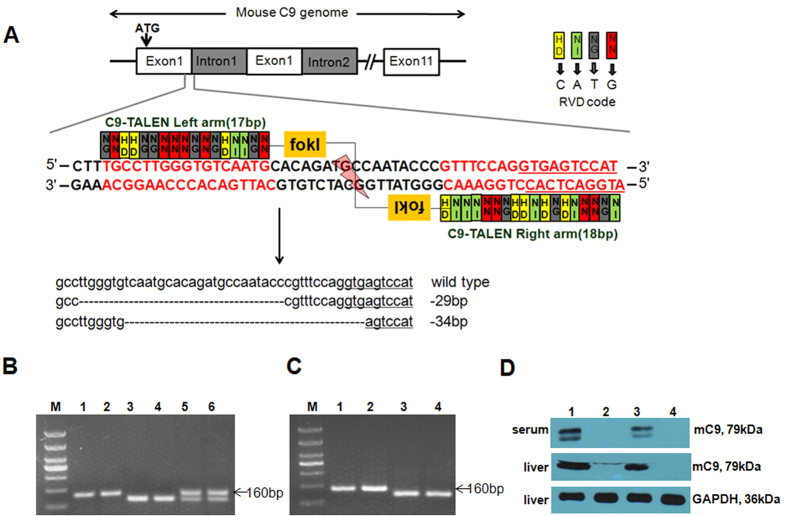
TALEN-mediated generation of *mC9*^−/−^ mice. (**A**) A schematic representation of the mouse C9 genome, the TALEN recognition site and the TALEN pairs used in this study. The top panel represents the genomic structure of the C9 gene. The middle panel indicates the target site, the TALEN pairs, and their binding site in exon 1 of the C9 gene. The bottom panel indicates the two types of deletions detected in the targeted region of the mouse C9 gene in embryos injected with C9 TALEN mRNA. (**B**) *mC9*^+/+^ (wild type, WT), *mC9*^+/−^ heterozygous, and *mC9*^−/−^ mice were genotyped with PCR method. A 160 bp band shown in Lane 1 and 2, 126 bp in Lane 3, 131 bp in lane 4, both 160 bp and 126 bp bands in lane 5, and both 160 bp and 131 bp bands in lane 6 indicate the mouse genotype for *mC9*^+/+^, *mC9*^−/−^ from founder 1 (−34 bp); *mC9*^−/−^ from founder 2 (−29 bp); *mC9*^+/−^ from founder 1, *mC9*^+/−^ from founder 2 (−29 bp), respectively. (**C**) Reverse-transcription PCR analysis of the total RNA from the liver tissues of *mC9*^+/+^ or *mC9*^−/−^ mice. Lane 1 and 2 show 160 bp RT-PCR products in the exon 1 of *mC9*^+/+^ mice. Lane 3 and lane 4 show 129 bp and 131 bp RT-PCR products in the exon 1 of *mC9*^−/−^ from founder 1 (−34 bp) and founder 2 (−29 bp), respectively. The primers used for genotyping PCR and RT-PCR are listed in the Methods section. (**D**) The level of C9 protein was detected by western blotting. The top, middle and bottom panels represent the levels of C9 protein in the serum, of C9 in whole cell extracts of the liver tissue, and GAPDH in whole cell extracts of the liver tissue. Lane 1, 2, 3, and 4 indicates the protein levels of *mC9*^+/+^, *mC9*^−/−^ from founder 1 (−34 bp), *mC9*^+/+^, and *mC9*^−/−^ from founder 2 (−29 bp), respectively.

**Figure 2:**
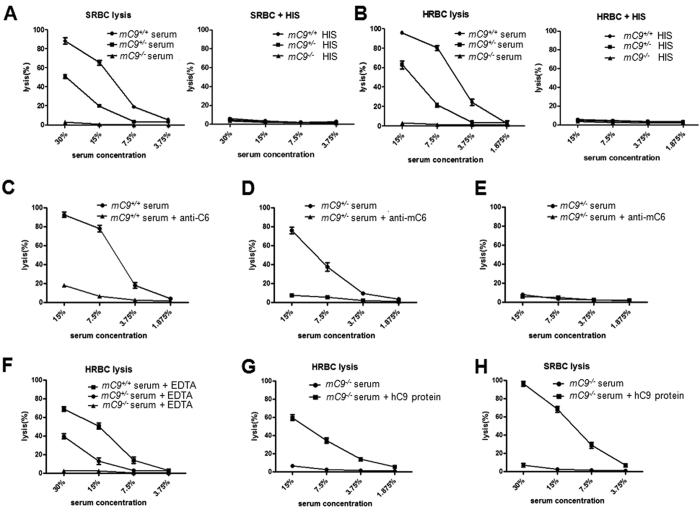
Deficiency in C9 attenuates complement-mediated hemolysis *in vitro*. (**A**) The capacity of *mC9*^+/+^ (n = 6)-, *mC9*^+/−^ (n = 6)- and *mC9*^−/−^ (n = 6)-dervied serum as a source of complement was tested in the complement-mediated hemolytic assay with rabbit poly-clonal anti-SRBC antibody-sensitized SRBCs or rabbit poly-clonal anti-HRBCs antibody-sensitized HRBCs. *mC9*^+/+^ *vs. mC9*^−/−^, *mC9*^+/+^ *vs. mC9*^+/−^, at serum concentrations of 30%, 15%, and 7.5%, P < 0.001; at serum concentration of 3.75%, P > 0.05; *mC9*^+/−^ *vs mC9*^−/−^ at serum concentrations of 30% and 15%, P < 0.001; at serum concentrations of 7.5% and 3.75%, P > 0.05. Heat-inactivated serum (HIS) from either *mC9*^+/+^, *mC9*^+/−^ or *mC9*^−/−^ animals as a source of complement did not have any hemolytic effect on antibody-sensitized SRBCs. (**B**) Complement-mediated hemolytic assay of HRBCs. *mC9*^+/+^ *vs. mC9*^−/−^, *mC9*^+/+^ *vs. mC*^+/−^, at serum concentrations of 15%, 7.5%, and 3.75%, P < 0.001; at serum concentration of 1.875%, P > 0.05; *mC9*^+/−^ *vs mC9*^−/−^, at serum concentrations of 15% and 7.5%, P < 0.001; at serum concentrations of 3.75% and 1.875%, P > 0.05. Heat-inactivated serum (HIS) from either *mC9*^+/+^, *mC9*^+/−^ or *mC9*^−/−^ animals as a source of complement did not have any hemolytic effect on antibody-sensitized HRBCs. (**C**) C6 deprivation attenuated the complement-dependent hemolysis of both SRBCs and HRBCs. (**D**) *mC9*^+/+^, *mC9*^+/−^ and *mC9*^−/−^ derived sera were pretreated with 100 μg/cm of the anti-mouse C6 neutralizing antibody and were then subjected to a hemolysis assay with SRBCs or HRBCs. C6 neutralization significantly downregulated the complement-mediated hemolysis in *mC9*^+/+^ and *mC9*^+/−^ serum, but not *mC9*^−/−^ serum. The results were representative of at least three repeated experiments. (**E**) The addition of C9 reconstituted the complement-mediated hemolytic capacity of *mC9*^−/−^ serum. EDTA-treated *mC9*^+/+^ or *mC9*^+/−^, but not *mC9*^−/−^, serum restored the complement-mediated hemolysis of *mC9*^−/−^ serum. The results were representative of at least three repeated experiments. *mC9*^+/+^: wild-type mice, SRBC: sheep red blood cells, and HRBC: human red blood cells. (G, H) Addition of recombinant human C9 protein (hC9) to the *mC9*^−/−^ serum reconstituted the hemolytic capacity of the *mC9*^−/−^ serum to SRBCs (G) and HRBCs (H).

To investigate the functionality of C5b-9 *in vivo*, we generated *mC9*^−/−^ mice by using a transcription activator-like effector nuclease (TALEN)-mediated targeted deletion strategy (Fig. 1A). We designed the target vector to delete exon 1 of the C9 genome because this exon encodes the signaling peptide of the murine C9 protein, and the interruption of exon 1 affects the secretion of C9 (Supplementary Fig. S1A). After screening 12 pairs of TALEN plasmids through restriction enzyme identification and sequencing, the pair 2L3/2R1, targeting the second site, was shown to have high TALEN activity (Supplementary Fig. S1B, Table S1). The mRNA of this pair was then injected into B6 background zygotes. We generated two founders (1 and 2), and genomic sequencing analysis demonstrated that founders 1 and 2 carried a −29 bp and a −34 bp deletion, respectively (Supplementary Fig. S1C). Both F0 founders were crossed with B6 mice to yield *mC9*^+/−^ F1 chimeras. Intercrossing the *mC9*^+/−^ mice generated *mC9*^−/−^, *mC9*^+/−^, and *mC9*^+/+^ offspring from each founder in the expected Mendelian ratio. The successful disruption of the *mC9* gene from each founder was confirmed by (1) genotyping the offspring through PCR by using specific primers (Fig. 1B), (2) reverse transcription PCR (RT-PCR) analysis with *mC9* specific primers (Fig. 1C), which showed the deletion of the expected 29 bp or 34 bp gene fragment in the *mC9* mRNA transcripts in liver tissue, and (3) DNA sequencing of the RT-PCR products (Supplementary Fig. S2B). From computer analysis of the normal and mutant transcripts of C9, the −29 bp or −34 bp deletions in exon 1 of the C9 mRNA were predicted to result in the early termination of protein translation at 124 bp or 136 bp, respectively, from the initiation codon (analyzed with ORF Finder from the NCBI website), thereby leading to a lack of the C9 protein in *mC9*^−/−^ mice. Consistent with this predication, western blotting analysis reveals that the *mC9*^−/−^ offspring from two founders but not *mC9*^+/+^ had not detectable C9 protein both serum and liver tissue (Fig. 1D, Supplementary Fig. S3). Phenotypically, we did not find any differences in body weight, fertility, gender (Supplementary Fig. S4A, S4B, and S4C), general activity and daily food intake between *mC9*^+/+^ and *mC9*^−/−^ mice.

### Deficiency of C9 attenuates MAC-mediated hemolysis *in vitro*

We further investigated whether the deletion in exon 1 of C9 in *mC9*^−/−^ mice led to a loss of C9 functionality. Considering that C9 is synthesized in the liver^25^, It been widely recognized that the mouse cells are resistant to mouse own complement-mediated lytic effect due to both the species specificity of complement regulation and the low complement activation in mouse serum as a source of complement^8^,^9^,^26^. Therefore, we tested the activity of serum C9 in the mice by using a complement-mediated hemolytic assay with antibody-sensitized sheep or human red blood cells (SRBCs or HRBCs, respectively) but not mouse RBC. The serum from the *mC9*^−/−^ offspring of the two founders carrying either the −29 bp or −34 bp targeted deletion as a source of complement did not cause significant hemolysis to either antibody-sensitized SRBCs or HRBCs (Supplementary Fig. S5A, S5B, S5C, and S5D) at any given concentration. These results indicate that the serum of mice derived from either of the founders showed complete loss of complement activity to mediate antibody-activated hemolysis. On the basis of these results, we used the offspring of the −34 bp founder as *mC9*^−/−^ mice to perform the following experiments. The mice carrying the −34 bp deletion showed a complete loss of complement activity and thus complement-dependent hemolysis either antibody-sensitized SRBCs or HRBCs (Fig. 2A,B). In contrast, both the *mC9*^+/+^ or wild-type (WT) and *mC9*^+/−^ sera mediated hemolysis in a dose-dependent manner. Moreover, the *mC9*^+/+^ serum demonstrated a higher hemolytic effect than the *mC9*^+/−^ serum (Fig. 2A,B), which indicates that C9 has a rate limit or dependent effect for the formation of MAC. This hemolytic effect was complement-dependent because heat-inactivated serum (HIS) from either *mC9*^+/+^ or *mC9*^+/−^ animals as a source of complement did not have any hemolytic effect on either antibody-sensitized SRBCs or HRBCs (Fig. 2A,B). These results indicate that *mC9*^−/−^ serum lacks complement activity for mediating the lysis of antibody-sensitized erythrocytes. In addition, both *mC9*^+/+^ and *mC9*^+/−^ serum pretreated with an anti-C6 neutralizing antibody (Ab) did not mediate the complement-dependent hemolysis of both types of RBCs (Fig. 2C,D). This result further confirms that the hemolytic effect is mediated by activating the terminal complement pathways. Moreover, *mC9*^−/−^ serum pre-treated with the anti-C6 Ab did not have any effect on *mC9*^−/−^ serum-mediated hemolysis (Fig. 2E). Furthermore, to document whether the lack of complement activity in *mC9*^−/−^ serum is a direct consequence of the specific deficiency of C9 in *mC9*^−/−^ serum, we performed a reconstitution experiment. Antibody-sensitized HRBCs were exposed to 15% *mC9*^−/−^ serum as a source of complement to form C5b-7 and C5b-8 on HRBCs. After the unlysed HRBCs were washed, they were exposed to 10 mM EDTA-pretreated *mC9*^+/+^, *mC9*^+/−^ or *mC9*^−/−^ serum as a source of C9. It is widely recognized that the removal of Ca^2+^ and Mg^2+^ from serum by using 10 mM EDTA pre-treatment blocks complement cascade activation because Ca^2+^ and Mg^2+^ are required for complement cascade activation^9^,^27^. As expected, both the EDTA-treated *mC9*^+/+^ and *mC9*^+/−^ sera, but not the *mC9*^−/−^ serum, clearly restored complement-mediated hemolysis of *mC9*^−/−^ serum in a concentration-dependent manner (Fig. 2F). Furthermore, the addition of 120 μg/ml recombinant human C9 (hC9) protein to *mC9*^−/−^ serum reconstituted complement mediated hemolytic capacity of *mC9*^−/−^ serum to lyse HRBCs (Fig. 2G) and SRBCs (Fig. 2H), respectively. Together, these data demonstrate that we successfully generated *mC9*^−/−^ mice.

### Deficiency in C9 attenuates complement-dependent hemolysis *in vivo*

**Figure 3:**
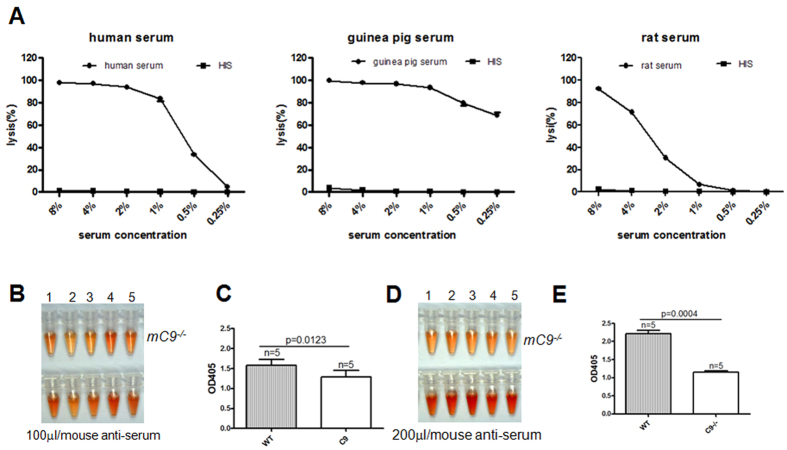
Deficiency in C9 attenuates complement-mediated hemolysis *in vivo*. (**A**) Serum dervied from different species contributed to different levels of hemolysis. Human-, guinea pig- and rat-derived sera were used as a source of complement to evaluate anti-MRBC antibody-mediated hemolysis. (**B**) Anti-murine RBC anti-serum induced lower levels of hemolysis *in vivo. mC9*^+/+^ (n = 5) and *mC9*^−/−^ (n = 5) mice received tail vein injections of 100 μl and 200 μl anti-serum. Twenty minutes later, the serum of each mouse was isolated for optical density detection at 450 nm or for image analysis.

We further investigated whether deficiency in C9 had any effect on complement-dependent hemolysis *in vivo*. First, we tested the efficacy of rabbit anti-mouse RBC poly-clonal antibodies (anti-MRBC) in activating complement-dependent hemolysis *in vitro* by using human, guinea pig or rat serum as a source of complement. As illustrated in Fig. 3A, serum mediated the complement-dependent hemolysis activated by the polyclonal antibody-sensitized MRBCs in a dose-dependent manner, although the rat serum showed the lowest hemolytic capacity compared with human or guinea pig serum. As a source of complement, heat-inactivated sera showed a complete loss of any hemolytic effect on MRBCs. These data demonstrated that the anti-MRBC anti-serum was capable of activating the complement present in serum. Second, we injected anti-MRBC anti-serum into the mice to evaluate antibody-activated complement hemolysis *in vivo. mC9*^+/+^ or *mC9*^−/−^ mice received two doses of the anti-serum (100 μl or 200 μl per mouse of anti-MRBC anti-serum injection). We found that, 20 min after tail vein injection with 100 μl or 200 μl anti-serum, *mC9*^+/+^ mice exhibited a significantly higher level of hemolysis than that seen in *mC9*^−/−^ mice (Fig. 3B–E). These results indicate that the deficiency of C9 in *mC9*^−/−^ mice attenuates complement-dependent hemolysis *in vivo*, which further suggests the successful generation of *mC9*^−/−^ mice.

### Deficiency in C9 protects against LPS-mediated septic acute shock

LPS is a well-known gram-negative bacterial membrane component that is responsible for bacterial-induced septic shock in both human and animals. It is well established that appropriate complement activation provides an important innate immune defense that protects the host against infection. However, inappropriate complement activation may also cause damage to the host. Emerging evidence has suggested that LPS-triggered uncontrolled systemic complement activation may lead to an irreversible state of septic shock^28^ by inducing cell death or a severe inflammatory reaction. However, the contribution of C5b-9 to LPS-induced shock remains to be determined.

**Figure 4:**
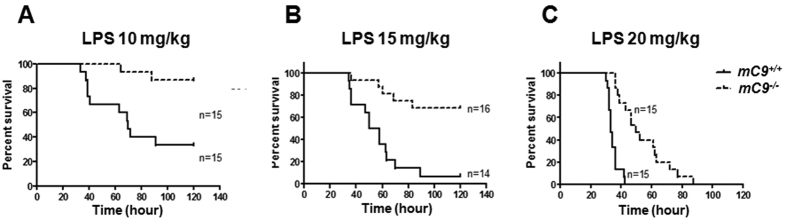
C9 deficiency protects mice against LPS-mediated septic acute shock. Eight- to ten-week-old (half male, half female) *mC9*^+/+^ and *mC9*^−/−^ mice received an i.p. injection of 10.0 mg/kg, 15.0 mg/kg or 20.0 mg/kg of LPS, and the survival of the mice was documented for up to 120 h. Animal survival was analyzed using a log-rank (Mantel-Cox) test. In mice treated with 10.0 mg/kg LPS via i.p. injection, *mC9*^+/+^ (n = 15) *vs. mC9*^−/−^ (n = 15), P = 0.020. The median survival time of *mC9*^+/+^ animals was 70 h, whereas that of *mC9*^−/−^ mice was undefined. With an i.p. injection of 15.0 mg/kg LPS, *mC9*^+/+^ (n = 14) *vs. mC9*^−/−^ (n = 16), P = 0.0020. The median survival time of *mC9*^+/+^ mice was 54 h, and that of *mC9*^−/−^ mice was undefined. With an i.p. injection of 20.0 mg/kg LPS, *mC9*^+/+^ (n = 15) *vs. mC9*^−/−^ (n = 15), P < 0.0001. The median survival time of *mC9*^+/+^ mice was 33 h, whereas that of *mC9*^−/−^ mice was 49 h.

*mC9*^+/+^ and *mC9*^−/−^ mice received an intraperitoneal (i.p.) injection of 10.0 mg/kg, 15.0 mg/kg or 20.0 mg/kg LPS per mouse, and the survival of the mice was recorded. Compared with *mC9*^+/+^ mice, *mC9*^−/−^ mice showed an increased survival rate for all the tested doses of LPS (Fig. 4A–C). As the LPS dose was increased, animal survival times were shortened, and the survival rates were reduced. However, the median survival times were significantly extended and the survival rates were reduced in *mC9*^−/−^ mice compared with *mC9*^+/+^ mice (Fig. 4A–C). These results collectively suggest that C9 deficiency renders mice resistant to LPS-induced shock and death.

### Deficiency in C9 results in the reduced release of cytokines associated with LPS-induced septic shock

One of the important mechanisms underlying LPS-induced septic shock is the severe inflammatory reaction triggered by LPS^29^. LPS initiates this inflammatory response, which may lead to host death, at least in part, through activation of the complement system. It is also well known that a sublytic complement complex and some bio-products of complement activation, such as C3a and C5a, contribute to promote inflammatory responses^30^,^31^. As such, it is conceivable that the resistance of *mC9*^−/−^ mice to LPS-induced shock might be associated with an attenuated inflammatory response. Multiple inflammatory cytokines have been suggested to promote LPS-induced inflammation. Among these, tumor necrosis factor alpha (TNFα) and IL-1β are thought to be critical for initiating an inflammatory response^29^. Therefore, their levels in the serum should reflect the level of inflammation *in vivo*.

**Figure 5:**
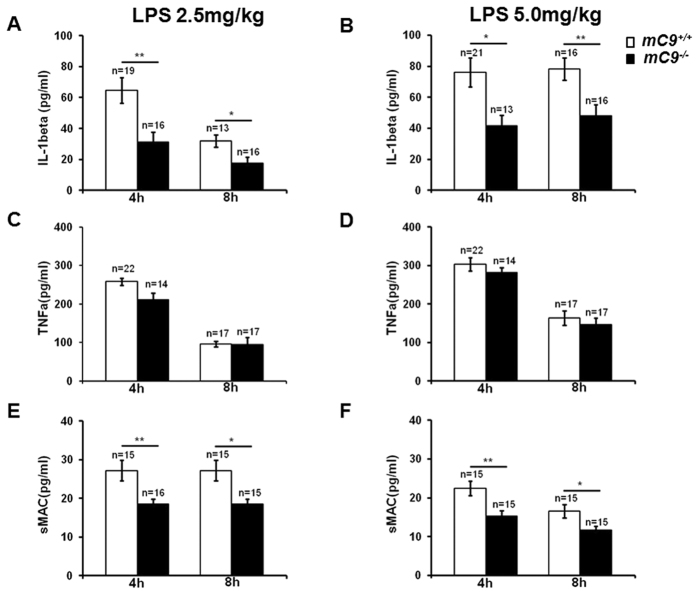
Decreased levels of IL-1β, TNFα and sMAC in *mC9*^−/−^ mice in response to LPS stimulation. *mC9*^+/+^ or *mC9*^−/−^ mice were administered LPS at a dose of 2.5 or 5.0 mg/kg/mouse, respectively. Mice were bled at 4 h and 8 h, and the sera were isolated for subsequent ELISA to detect circulating TNFα (**A**,**B**), IL-1β (**C**,**D**) and sMAC (**E**,**F**). After a 2.5 mg/kg LPS challenge, **P < 0.01 *mC9*^+/+^ vs. *mC9*^−/−^ for IL-1β (P = 0.0068) and sMAC (P = 0.0061) at 4 h. *P < 0.05 *mC9*^+/+^ vs. *mC9*^−/−^ for IL-1β (P = 0.0123) and sMAC (P = 0.0206) at 8 h. P > 0.05 for TNFα at any time point. After a 5.0 mg/kg LPS challenge, **P < 0.01 *mC9*^+/+^ vs. *mC9*^−/−^ for IL-1β (P = 0.0056) at 8 h and sMAC (P = 0.0052) at 4 h. *P < 0.05 *mC9*^+/+^ vs. *mC9*^−/−^ for IL-1β (P = 0.0120) at 4 h and sMAC (P = 0.0247) at 8 h. P > 0.05 for TNFα at any time point. The data were analyzed using an unpaired t test (two-tailed).

To investigate the inflammatory responses in *mC9*^+/+^ or *mC9*^−/−^ mice, the mice were treated with two different non-lethal doses of LPS (2.5 mg/kg and 5.0 mg/kg). Mice were bled at 4 h and 8 h after LPS treatment, and the sera were analyzed with an enzyme-linked immunosorbent assay (ELISA) to detect circulating TNFα and IL-1β. The levels of TNFα in both groups peaked 4 h after LPS injection and quickly decreased by approximately half after 8 h. We found that *mC9*^−/−^ mice showed no difference in the levels of serum TNFα at either 4 h or 8 h after LPS injection, as compared with *mC9*^+/+^ mice (Fig. 5A,B). Importantly, the serum IL-1β level peaked at 4 h but only slightly declined at 8 h, and *mC9*^−/−^ mice showed dramatically lower levels of serum IL-1β at both 4 h and 8 h after LPS injection, as compared with *mC9*^+/+^ mice (Fig. 5C,D). This result suggests that C5b-9 has a profound effect on LPS-induced IL-1β release. Furthermore, we measured the level of soluble C5b-9, indicative of C5b-9 formation, in the mice after LPS treatment. We found that sC5b-9 peaked at 4 h and declined at 8 h in both groups, and *mC9*^−/−^ mice showed a significantly lower sC5b-9 concentration than *mC9*^−/−^ mice (Fig. 5E,F). The high background detection in *mC9*^−/−^ mice may result from the specific recognition of serum C5b, C6, C7 and C8 by rabbit poly-clonal anti-mouse MAC antibodies used here since those anti-MAC Abs were produced using mouse MAC as antigen. These results further show that C5b-9 has a more profound effect on the release of IL-1β than TNFα, which may explain the attenuation of the LPS-induced inflammatory effects observed in *mC9*^−/−^ mice (Fig. 5).

### Deficiency of C9 suppresses caspase-1 activation and reduces IL-1β secretion after LPS treatment

We further explored the underlying mechanism by which the MAC leads to a profound effect on IL-1β. IL-1β belongs to the non-classical pathway of secreted proteins, because its gene lacks a signal peptide sequence. In most cases, the maturation and release of IL-1β depends on the formation of a multiple-protein complex known as the inflammasome^32^. To date, at least four types of inflammasomes, including NLRP1, NLRP3, NLRC4, and AIM2, have been reported to process the pro-protein of IL-β (pro-IL-1β) into secreted mature IL-1β (sIL-1β). These inflammasomes contain multiple proteins, including caspase-1, which is well recognized for its processing of pro-IL-1β into the secreted form^33^. Accordingly, inhibition of caspase-1 has been shown to inhibit IL-1β secretion^34^,^35^.

**Figure 6:**
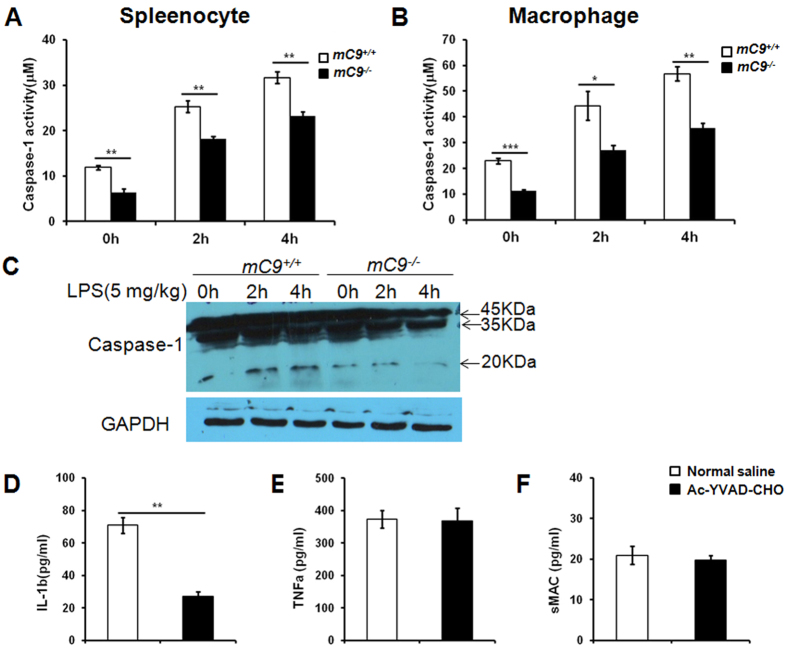
Decreased caspase-1 activation in *mC9*^−/−^ mice after LPS treatment. *mC9*^+/+^ or *mC9*^−/−^ mice were injected i.p. with 5.0 mg/kg per mouse of LPS for 2 h or 4 h. Whole-cell extracts from splenocytes (**A**) and peritoneal macrophages (**B**) were used for caspase-1 activity detection. A total of 100 μg of the whole cell extract from splenocytes was separated on a 15% SDS-PAGE gel and then subjected to western blotting to detect the activation of caspase-1 (**C**). *mC9*^+/+^ mice were pretreated with an i.p. injection of 5.0 mg/kg of the caspase-1 specific inhibitor Ac-YVAD-CHO before a 5.0 mg/kg per mouse LPS challenge. The circulating levels of IL-1β (**D**), TNFα (**E**) and sMAC (**F**) were detected 4 h after LPS stimulation. In the splenocyte caspase-1 activity assay, **P < 0.01 vs. *mC9*^+/+^ at any detected time point with P = 0.0047, P = 0.0069, and P = 0.0070 at 0 h, 2 h, and 4 h, respectively. In the macrophage caspase-1 activity assay, ***P < 0.001 vs. *mC9*^+/+^ at 0 h (P = 0.0008), *P < 0.05 vs. *mC9*^+/+^ at 2 h (P = 0.0436), and **P < 0.01 vs. *mC9*^+/+^ at 4 h (P = 0.0033). For the Ac-YVAD-CHO treatment, **P < 0.01 vs. *mC9*^+/+^ for IL-1β (P = 0.0014), P > 0.05 vs. *mC9*^+/+^ for TNFα (P = 0.8705) and sMAC (P = 0.4644). The data were analyzed using an unpaired t test (two-tailed).

Therefore, we used total splenocytes and peritoneal macrophages to investigate whether decreased MAC formation because of C9 deficiency had any effect on caspase-1 activation and the subsequent release of IL-1β. After the mice were treated with non-lethal doses of LPS (5.0 mg/kg per mouse LPS) for 2 h or 4 h, the cells were prepared, and whole cell extracts were used for caspase activity detection. Our data showed that LPS induced a significantly higher level of caspase-1 activity (Fig. 6A,B) in m*C9*^+/+^ splenocytes compared with that in *mC9*^−/−^ splenocytes. We also observed markedly increased caspase-1 activation in the splenocytes of m*C9*^+/+^ mice but not those of *mC9*^−/−^ mice (Fig. 6C, Supplementary Fig. S6A, S6B and S6C). Furthermore, to evaluate whether the enhanced level of IL-1β was dependent on caspase-1 activation, m*C9*^+/+^ mice were pretreated with Ac-YVAD-CHO, a caspase-1 specific inhibitor^36^ (i.p. 5.0 mg/kg per mouse for 1 h), and then the mice were treated with a non-lethal dose of LPS (i.p. 5.0 mg/kg) for 2 h or 4 h. The Ac-YVAD-CHO injection decreased the serum levels of IL-1β but not TNFα (Fig. 6D–F). Together, these data suggest that C5b-9 contributes to LPS-induced IL-1β secretion in a caspase-1-dependent manner.

## Discussion

Here, we report the successful generation of *mC9*^−/−^ mice and demonstrate that the lack of C9 attenuates anti-erythrocyte antibody-mediated hemolysis *in vitro* and *in vivo*. These results indicate that the C5b-9 complex is the most important among the terminal complement complexes. Over the years, it has been established that the complement system has versatile functions and that all of the complement cascades activated by the three different complement pathways converge in a common terminal pathway. In this pathway, multiple complement components, including the C5b, C6, C7, C8, and C9 proteins, form several sequential complexes, such as C5b-7, C5b-8, and C5b-9. The binding of the C5b-8 complex to C9 triggers the polymerization of C9, leading to formation of the MAC. The C5b-9 complex forms a lytic pore in the lipid bilayer of the membrane, which results in the killing of foreign pathogens and infected host cells^1^,^2^,^3^,^4^. Our results reported herein further confirm this notion.

On the basis of the lytic capacity of C5b-9 (or MAC), the MAC can be classified as lytic or sublytic^37^. The sublytic MAC, which often forms on nucleated cells, does not kill the target but triggers diverse effects in different types of cells, including the activation of signaling pathways, the release of inflammatory mediators and the promotion or inhibition of cell proliferation and apoptosis^38^,^39^. In addition to the C5b-9 complex, the C5b-7 and C5b-8 complexes are also able to form nonlytic pores, thereby mediating the activation of signaling pathways^19^,^21^. Although multiple downstream signaling pathways have been implicated in the effects of the sublytic MAC, no consensus signaling cascades have been found^13^. Several reports from different laboratories have ascribed pro-inflammatory consequences to the sublytic MAC. These studies have indicated that the sublytic MAC is able to trigger neutrophils and macrophages^37^, mesangial cells and microglia, and retinal epithelial cells^40^ to release inflammatory mediators^41^,^42^. Thus, these data identify the MAC as an important driver of inflammation. Most recently, several publications have linked complement-mediated inflammation to inflammasome activation and IL-1β secretion. Cholesterol crystals, through the complement system, promote LPS-mediated inflammasome/caspase-1 activation, which leads to the release of mature IL-1β^18^. In addition, Triantafilou *et al*. have found that deposition of the sublytic MAC on the cell surface leads to the assembly and activation of the NLRP3 inflammasome as well as the release of IL-1β in LPS-primed lung epithelial cells. These authors have also reported that by increasing Ca^2+^ influx via the MAC pore and releasing Ca^2+^ from intracellular stores, the sublytic MAC triggers mitochondrial damage and cellular apoptosis^43^. Together, these results show that the sublytic MAC triggers inflammation *in vivo* primarily through NLRP3 inflammasome activation and IL-1β secretion. Interestingly, in that study, the authors also used C6-deficient mice to evaluate the role of the MAC *in vivo*. They have found that MAC deposition is impaired in C6-deficient mice and that deficiency in C6 (inability to generate C5b-7, C5b-8, and C5b-9) significantly reduces the serum IL-1β levels, whereas the IL-6 levels remain unchanged. These data support the notion that the sublytic MAC triggers inflammation *in vivo* primarily through inflammasome activation^17^. These findings also suggest a combinational effect of the C5b-7, C5b-8 and C5b-9 complexes, because C6 deficiency affects the entire complement activation cascade but not other terminal complement complexes. However, the roles of these complexes in inflammatory responses in a disease setting remain unclear.

In the current study, we sought to evaluate the role of the sublytic MAC *in vivo* and extend the results of previous studies to a pathological setting, specifically LPS-induced shock in *mC9*^−/−^ mice. We found that C9 deficiency significantly protected mice against LPS-induced shock. C9 deficiency also led to decreased MAC formation and dramatically lowered levels of serum IL-1β after LPS treatment. Our data also showed that the sublytic MAC triggered the release of IL-1β, but not TNFα, in a manner dependent on the activation of caspase-1. Furthermore, the MAC has a more profound effect on the release of IL-1β than on TNFα, which may explain the attenuated LPS-induced inflammatory effects observed in *mC9*^−/−^ mice. In the present research, we found that the C5b-9 complex is the primary inflammatory trigger of the LPS-induced inflammatory reaction. However, there remain questions that should be addressed in future studies; in particular, the underlying signaling cascades triggered by the MAC and the upstream inflammasome responsible for caspase-1 activation and IL-1β release remain unknown.

In addition, although the MAC provides an important defense mechanism against foreign pathogens in the host, this complex can also attack host cells (autologous cells), leading to a variety of cellular injuries^44^. The classical notion is that the complete C5b-9 MAC is important in stimulating the lysis of target cells, either through the induction of necrosis or apoptosis^45^. This mechanism is thought to be critical for the clearance of microbial pathogens, especially Gram-negative bacteria in humans. In the past 40 years, the importance of C9 to the complement response has been addressed in C9-deficient patients, because deficiencies in C9 have been shown to be associated with recurrent invasive infections because of inefficient MAC formation. Fine *et al*. first reported the association of C9 deficiency and disseminated acute *Neisseria meningitidis* in a 17-year-old woman^46^. Subsequently, more clinical data have been reported on the association of C9 deficiency with recurrent *Neisseria meningitides* infection^47^,^48^. Together, these data strongly support the notion that C9 is involved in achieving the bactericidal function of the MAC and that the involvement of C9 is important in the defense against infections. Although it is now known that C9 contributes to bacterial elimination, in the *mC9*^−/−^ model we presented here, we addressed the contribution of C9 to inflammation in the form of LPS-induced shock. Indeed, our findings indicate that LPS, through induction of the sublytic MAC, might trigger or accelerate LPS-induced shock. These data help to establish the biological functions of C9 and the MAC under physiological and pathological conditions.

Furthermore, the generation of *mC9*^−/−^ mice will further facilitate understanding of the role of the MAC in the pathogenesis of complement-related diseases. The contribution of the sublytic MAC to the pathogenesis of human diseases has been extensively investigated by using C5, C6 and CD59 knockout animals. For example, in C5- and C6-deficient mice, it has been found that the MAC contributes to multiple diseases, including seizures in an experimental cerebral malaria model^49^, brain ischemia^50^, renal ischemia/reperfusion injury^51^, atherosclerosis^52^, and Ab-mediated antiphospholipid syndrome (APS)^53^. Using CD59-deficient mice, we^54^,^55^,^56^ and others^52^,^57^,^58^ have demonstrated that the MAC contributes to the development of atherosclerosis and aneurysms. Collectively, these data elucidate the critical role of the MAC in the development of disease. Nevertheless, because the deletion of C5, C6 or CD59 will affect the entire terminal complement pathway, the roles of the MAC identified in these studies represent the combinational effects of C5b-7, C5b-8 and C5b-9. To clearly understand the critical role of C5b-9 in these diseases, we successfully generated a *mC9*^−/−^ mouse model by using a TALEN targeted deletion approach. Therefore, our *mC9*^−/−^ mice provide a new tool to further dissect the function of the terminal complement pathway in pathological and physiological conditions.

## Methods

### Animals

Animal studies were conducted according to protocols approved by the Weifang Medical University Institutional Animal Care and Use Committee.

### Reagents and antibodies

We used the following reagents and antibodies for our studies: LPS purchased from Sigma-Aldrich (*Escherichia coli* 0111:B4), a rabbit anti - mouse C6 poly–clonal antibody (Biobyt, Berkeley, CA, USA), a rabbit anti-caspase-1 polyclonal antibody (ProteinTech, Chicago, IL, USA), a rabbit anti-C9 polyclonal antibody (Cloud-clone Corp, Huston, TX, USA), an anti-sMAC ELISA kit (Miobiosource, San Diego, CA, USA), a colorimetric caspase-1 activity kit (Biovision, Milpitas, CA, USA), a RIPA lysis buffer (Applygen, Beijing, China), the caspase-1 specific inhibitor Ac-YVAD-CHO (Merk, Kenilworth, NJ, USA), an enhanced chemiluminescence (ECL) kit (Pierce, Rockford, lL, USA), a rabbit anti-mouse GAPDH antibody, a horseradish peroxidase (HRP)-conjugated goat anti-rabbit antibody and whole cell lysis buffer for western blotting (Cell Signaling Technology, Danvers, MA, UK). The protein A/G agarose (Thermo Scientific, Rockford, lL, USA), the recombinant human C9 (hC9) protein (Complement Technology, Tyler, TX, USA), anti-IL-1β and anti-TNFα Ready-SET-Go! Sandwich ELISA kits were also used (eBioscience, San Diego, CA, USA).

#### Generation ofh mC9^−/−^ mice

*mC9*^−/−^ mice were constructed by using a TALEN-mediated strategy (Si-dansai Biotechnology, Shanghai, China). Briefly, according to the mouse C9 gene, 12 TALEN pairs in the form of 2 × 3 TALEN combinations were designed targeting two different sites (Supplementary Fig. S1A, Supplementary Table S1). The TALEN pairs were transfected into NIH3T3 mouse cells, and then puromycin screening was performed. The genomic DNA extracted from NIH3T3 cells was used for PCR amplification (sense primer: 5′-GCATAATGACACTTTGTCAATAAGG-3′, antisense primer 5′-CTTAGCATTAACATTTCAGTCCCTC-3′) to verify the activity of the TALEN plasmids. Finally, one pair of TALEN plasmids (left arm 2L3 and right arm 2R1) targeting the second site was shown to have high activity, because PCR product sequencing showed a clear peak (Supplementary Fig. S1B). The TALEN mRNAs with the highest activity were injected into zygotes to obtain F0 mutants. After genotyping, we identified two *mC9*^+/−^ propositas with either a 34 bp (−34 bp) or 29 bp (−29 bp) deletion in the *C9* genome (Supplementary Fig. S1C). The F0 founders were hybridized with *mC9*^+/+^ B6 background mice to obtain the F1 generation of heterozygous mice. The F1 *mC9*^+/−^ mice were intercrossed to generate *mC9*^−/−^, *mC9*^+/−^, and *mC9*^+/+^ offspring from each founder.

#### Measurement of complement activity with a hemolytic assay *in vitro*

To measure complement activity, SRBCs and HRBCs were used to perform a hemolytic assay. SRBCs or HRBCs were washed and suspended in Gelatin Veronal buffer containing Mg^2+^ and Ca^2+^ (GVB++). A 1% SRBCs or HRBCs suspension was incubated for 30 min at 37 °C with 1/200 diluted rabbit anti-SRBC or rabbit anti-HRBC serum. The Ab-sensitized erythrocytes were washed and re-suspended in GVB++ to 2%. Then, 100 μl of the erythrocyte suspension with GVB++ diluted with an equal volume of serum (to lyse the SRBCs, the serum was diluted to 30%, 15%, 7.5%, and 3.75%; to lyse the HRBCs, the freshly prepared mouse serum was diluted at 15%, 7.5%, 3.75%, 1.875%) from *mC9*^+/+^, *mC9*^+/−^ or *mC9*^−/−^ mice was added to 96-well plates in triplicate. The fresh mouse serum as a source of complement was prepared as the following procedure. We harvested the blood from the mice and keep it in room temperature for 20–25 min for clotting followed by centrifuging 4,000 rpm at 4 °C for 5 min, the sera was collected and immediately used for the hemolytic assay.

The plates were incubated for 30 min at 37 °C. Unlysed cells were removed by centrifugation, and hemoglobin in the supernatant was measured on the basis of the OD_450_. The percentage lysis was calculated as follows: % LYSIS = (test OD_450_ − blank OD_450_/total lysis OD_450_) × 100.

To deplete C6 and upper level complement activation, *mC9*^+/+^, *mC9*^+/−^ or *mC9*^−/−^ serum was incubated with 100 μg/ml of rabbit anti-mouse C6 polyclonal antibody (Biobyt, orb182513) for 30 min at 37 °C before the hemolysis reactions.

To reconstitute the complement-mediated hemolysis caused by the *C9*^−/−^ serum, Ab - sensitized HRBCs were incubated primarily with GVB++ - diluted 15% *mC9*^−/−^ derived serum. The unlysed erythrocytes were washed and collected by centrifugation. A 2% erythrocyte suspension was prepared by adding normal saline (NS) to the HRBC pellet. *mC9*^+/+^, *mC9*^+/−^ or *mC9*^−/−^ serum was pretreated with 10 mM EDTA for 30 min and then diluted with NS before addition of an equal volume to the erythrocyte suspension. Hemoglobin in the supernatant and the percentage lysis were analyzed as described above.

To confirm whether the anti-serum-induced hemolysis was complement-dependent, we also used different species-derived GVB++-diluted serum from human (4%, 2%, 1%, and 0.5%), sheep (4%, 2%, 1%, and 0.5%) and rat (8%, 4%, 2%, and 1%) to hemolyse antibody-sensitized erythrocytes from *mC9*^+/+^ mice.

#### Preparation of rabbit anti-sheep, anti-human and anti-mouse erythrocyte anti-sera

The anti-RBC anti-serum to either SRBCs, HRBCs or mouse red blood cells (MRBCs) was kindly provided by the Nuoanke Biotechnology Company, Ltd. (Weifang, Shangdong, P. R. China). Briefly, erythrocytes were purified from peripheral blood. The RBCs were washed and resuspended in NS. Then, 1.0 ml of 20% (v/v) erythrocytes was injected subcutaneously into the back of each rabbit once per week for up to 2 months. The serum was isolated, and the titer of anti-serum against different types of RBCs was determined through an RBC agglutination assay. The titers of the anti-SRBC, anti-HRBC and anti-MRBC serum were 1:2,000, 1:1,000 and 1:1,000, respectively.

#### Measurement of complement activity with the hemolytic assay *in vivo*

To detect *in vivo* hemolysis induced by anti-MRBC anti-serum, male *mC9*^+/+^or C9^−/−^ mice were injected with 100 μl, 200 μl, or 400 μl rabbit anti-MRBC anti-serum through the tail vein for 20 min (n = 5 for each group). Then, 100 μl of serum was used to detect the hemoglobin by reading the optical density spectrophotometrically at 450 nm.

#### Mice treated with LPS

Eight- to ten-week-old male or female (each comprising half the study sample) *mC9*^+/+^ and *mC9*^−/−^ mice were injected i.p. with 10.0 mg/kg, 15.0 mg/kg, or 20.0 mg/kg LPS dissolved in NS. The survival of mice was monitored hourly for up to 120 hours. The survival curves were plotted by the Kaplan-Meier method and compared by using the log-rank test.

To assess the level of the inflammatory cytokines IL-1β and TNFα as well as soluble MAC (sMAC), mice were injected i.p. with 2.5 mg/kg, 5.0 mg/kg LPS or an equal volume of NS. At 4 h and 8 h after the injection, the serum was prepared for ELISA analysis. To detect caspase-1 activation, the splenocytes and peritoneal macrophages were obtained from mice 2 h and 4 h after i.p. injection of 2.5 mg/kg or 5.0 mg/kg LPS.

#### ELISA detection of IL-1β, TNFα, and sMAC

At 4 h or 8 h after i.p. injection of 5.0 mg/kg or 5.0 mg/kg LPS, the serum was isolated from *mC9*^+/+^ or C9^−/−^ mice. The levels of IL-1β and TNFα were detected with a Ready-SET-Go! kit. The level of sMAC was determined by captured rabbit poly-clonal anti-MAC Abs and detected rabbit poly-clonal anti-MAC-abs conjugated with biotin with a sandwich ELISA kit according to the manufacturer’s instructions.

#### PCR, reverse-transcription (RT) PCR and RT-PCR sequencing

Genomic DNA was extracted from the tail with tail lysis buffer. Total RNA was extracted from liver tissue with a TRIzol RNA isolation kit (Invitrogen), and these RNAs were used in PCR or RT-PCR to screen the *mC9*^−/−^ founders. The primer pairs used for PCR detection in the *mC9*^−/−^(−34 bp and −29 bp) founders were the sense primer, 5′-CCATCACCTTAGCCCTTGCCATCT-3′, and the antisense primer, 5′-CTTAACCTTTATTGTCCCTACTTTG-3′. The expected DNA product had a size of 160 bp for *mC9*^+/+^, 126 bp for *mC9*^−/−^ founder 1 (−34 bp) and 131 bp for *mC9*^−/−^ founder 2 (−29 bp). Primer pairs for the RT-PCR analysis included the sense primer 5′-GTCCTCCGGCTGCAAAGGAATGC-3′ and the antisense primer 5′-GTCTATCGGTATGGGATAGTGTT-3′. The expected product had a size of 160 bp for *mC9*^+/+^ and 129 bp for *mC9*^−/−^ founder 1 (−34 bp) and 131 bp for *mC9*^−/−^ founder 2 (−29 bp). For DNA sequencing, the purified RT-PCR product was ligated into the pMD-18T simple vector and sent for sequencing.

#### Caspase-1 activation and inhibition assay

The splenocytes of *mC9*^+/+^ or *mC9*^−/−^ mice treated with 5.0 mg/kg LPS for 2 h or 4 h were isolated and a total 10^7^ cells were lysed in 100 μl cell lysis buffer (provided with the kit) on ice for 30 min. After centrifugation, the cell lysates (100 μg total protein per sample) were separated on 15% SDS-PAGE gel and were then subjected to western blotting. The antibodies used included: rabbit anti-caspase-1 poly-clonal antibody (1:1,000 dilution), rabbit anti-GAPDH antibody (1:1,000 dilution) and HRP-conjugated goat anti-rabbit poly-clonal antibody (1:2,000 dilution).

Caspase-1 activity was measured using a colorimetric caspase-1 activity assay kit. A total of 100 μg of protein per sample from splenocytes or macrophages was incubated with 200 μM of substrate (Ac-YVAD-pNA) at different times as indicated by the manufacturer. The absorbance was quantified by using a microtiter plate reader at 405 nm. For caspase-1 inhibition, mice were administered i.p. 5.0 mg/kg caspase-1 specific inhibitor (Ac-YVAD-CHO) for 1 h prior to 5.0 mg/kg LPS stimulation. All experiments were repeated at least three times.

#### Immunoprecipitation (IP) of C9 protein

300 μl serum from either *mC9*^+/+^ or *mC9*^−/−^mice was precleared by incubating with 20 μl protein A/G agarose at 4 °C for 2 h. After centrifuging at 2,500 rpm for 5 min, the supernatant was incubated overnight at 4 °C with 2 μg rabbit anti-mouse C9 poly - clonal antibody. After washing 5 times with PBS, the immunoprecipitated proteins were eluted from the protein A/G agarose by boiling in 100 μl SDS-PAGE sample buffer. 10 μl of each sample was then subjected to SDS-PAGE and western blotting with anti – C9 antibody.

#### Western blotting detection of C9 protein

A total of 1 mg liver tissue was lyszed in 800 μl of RIPA lysis buffer on ice for 30 min. After centrifugation, 40 μg total protein per sample was separated on 10% SDS-PAGE gel and was then subjected to western blotting. To detect C9 level in the serum of *mC9*^−/−^ and *mC9*^+/+^ mice, 10 μl of IP products from each sample were used. The antibodies used included: rabbit anti – mouse C9 poly - clonal antibody (1:200 dilution), rabbit anti – mouse GAPDH antibody (1:1,000 dilution) and HRP – conjugated goat anti – rabbit polyclonal antibody (1:2,000 dilution).

### Statistical analysis

Differences between two groups were compared using a 2-tailed Student’s *t* test for unpaired data. Differences among multiple groups were analyzed with a two-way analysis of variance (ANOVA) using Prism software. The data are presented as the mean ±SEM. In all cases, P < 0.05 was considered to be statistically significant.

## Additional Information

**How to cite this article**: Fu, X. *et al*. Target deletion of complement component 9 attenuates antibody-mediated hemolysis and lipopolysaccharides (LPS)-induced acute shock in mice. *Sci. Rep.* **6**, 30239; doi: 10.1038/srep30239 (2016).

## Supplementary Material

Supplementary Information
