# Supplementary material for: Target deletion of complement component 9 attenuates antibody-mediated hemolysis and lipopolysaccharide (LPS)-induced acute shock in mice
Source: Sci Rep. 2016 Jul 22;6:30239. doi: 10.1038/srep30239 (PMC4957234; doi:10.1038/srep30239)
Supplement: Supplementary Information [file srep30239-s1.doc]

**Target deletion of complement component 9 attenuates antibody-mediated hemolysis and lipopolysaccharides (LPS)-induced acute shock in mice**

Xiaoyan Fu^1#^, Jiyu Ju^1#^, Zhijuan Lin^1^, Weiling Xiao^1^, Xiaofang Li^1^, Baoxiang Zhuang^1^,Tingting Zhang^1^, Xiaojun Ma^1^, Xiangyu Li^1^, Chao Ma^1^, Weiliang Su^1^, Yuqi, Wang^1^, Xuebin Qin^2^*, Shujuan Liang^1^*

^1^Key Lab for Immunology in Universities of Shandong Province, School of Clinical Medicine, Weifang Medical University, Weifang, 261053, P.R.China.

^2^Department of Neuroscience, Temple University School of Medicine, Philadelphia, PA19140, USA.

^#^ Co-first author, these authors contributed equally to this work

*Corresponding author: Xuebin Qin, M.D., Ph.D., Department of Neuroscience, Temple University School of Medicine, Room 749, 3500 N Broad Street, Philadelphia, PA 19140, Tel: 215-707-5823, Email: [xuebin.qin@temple.edu](mailto:xuebin.qin@temple.edu). Shujuan Liang, M.D., Ph.D., Key Lab for Immunology in Universities of Shandong Province, School of Clinical Medicine, Weifang Medical University, 7166 West Baotong Street, Weifang, 261053, P.R.China. Tel: 86-536-8462468, Email: [liangshj@wfmc.edu.cn](mailto:liangshj@wfmc.edu.cn).

**Supplementary data:**

**Method:**

**Bleeding time:** Six to eight – week old (half male and half female) mice were placed inside a restrainer and a distal tail tip (approximately 0.5 cm) was briskly cut at time 0 using a sharp new scalpel blade. The tail was immediately dipped into pre – warmed (37℃) PBS (2 cm under the water), the time until the bleeding stopped was recorded.

**Supplementary Figures:**

**
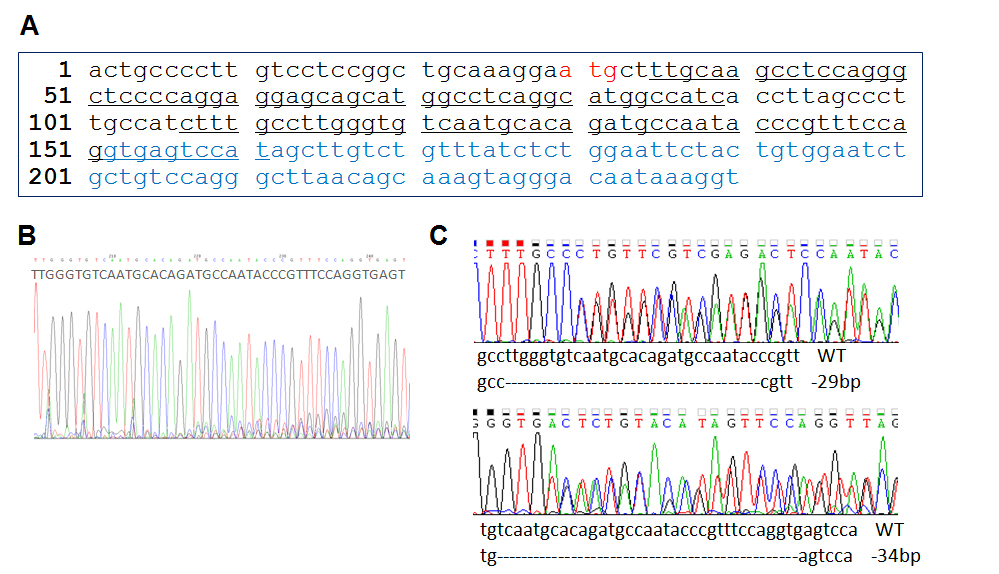
**

**Supplementary Figure 1:** (**A**) Schematic representation of the two target sites for TALEN mediated mouse *C9* gene knockout. Underlined were two target sequences. Exon 1 of *C9* was shown in black font, partial of intron 1 was shown in blue font. Initiation codon ATG was shown in red font. Signaling peptide coding sequence locates from 30 to 128 bp in exon 1. (**B**) PCR product sequencing proved that the left arm 2L3 and right arm 2R1 pair of TALEN plasmids had high activity as this pair showed obvious peak during sequencing. (**C**) Genotyping identified two heterozygous founders as the -29 bp and -34 bp F0 mutants. Primer pairs used for TALEN activity assay and genotyping PCR: sense primer 5´ - GCATAATGACACTTTG TCAATAAGG - 3´, antisense primer 5´ - CTTAGCATTAACATTTCAGTCCCTC - 3´.

**
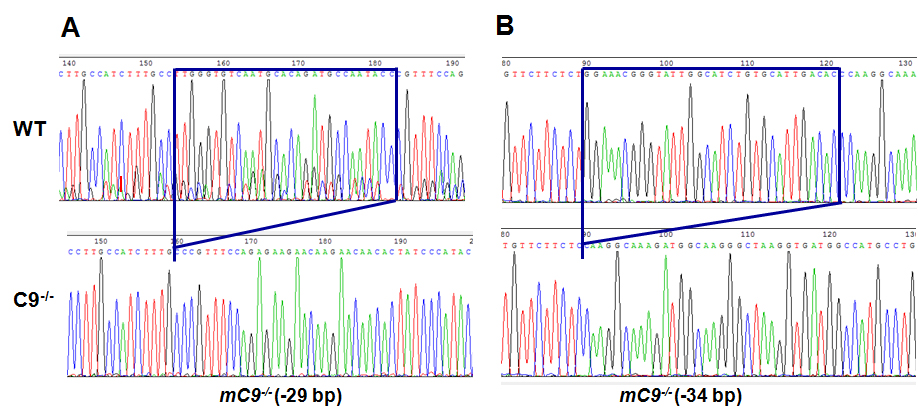
**

**Supplementary Figure 2:** DNA sequencing of reverse transcription PCR (RT-PCR) products identified two *mC9^-/-^* homozygous founders with either a -34 bp (**A**) or -29 bp (**B**) deletion respectively. Primer pairs used for RT-PCR were: sense primer 5' - GTCCTCCGGCTGCAAAGGAATGC - 3`, antisense primer 5' - GTCTATCGGTATGGGATAGTGTT - 3'.

**
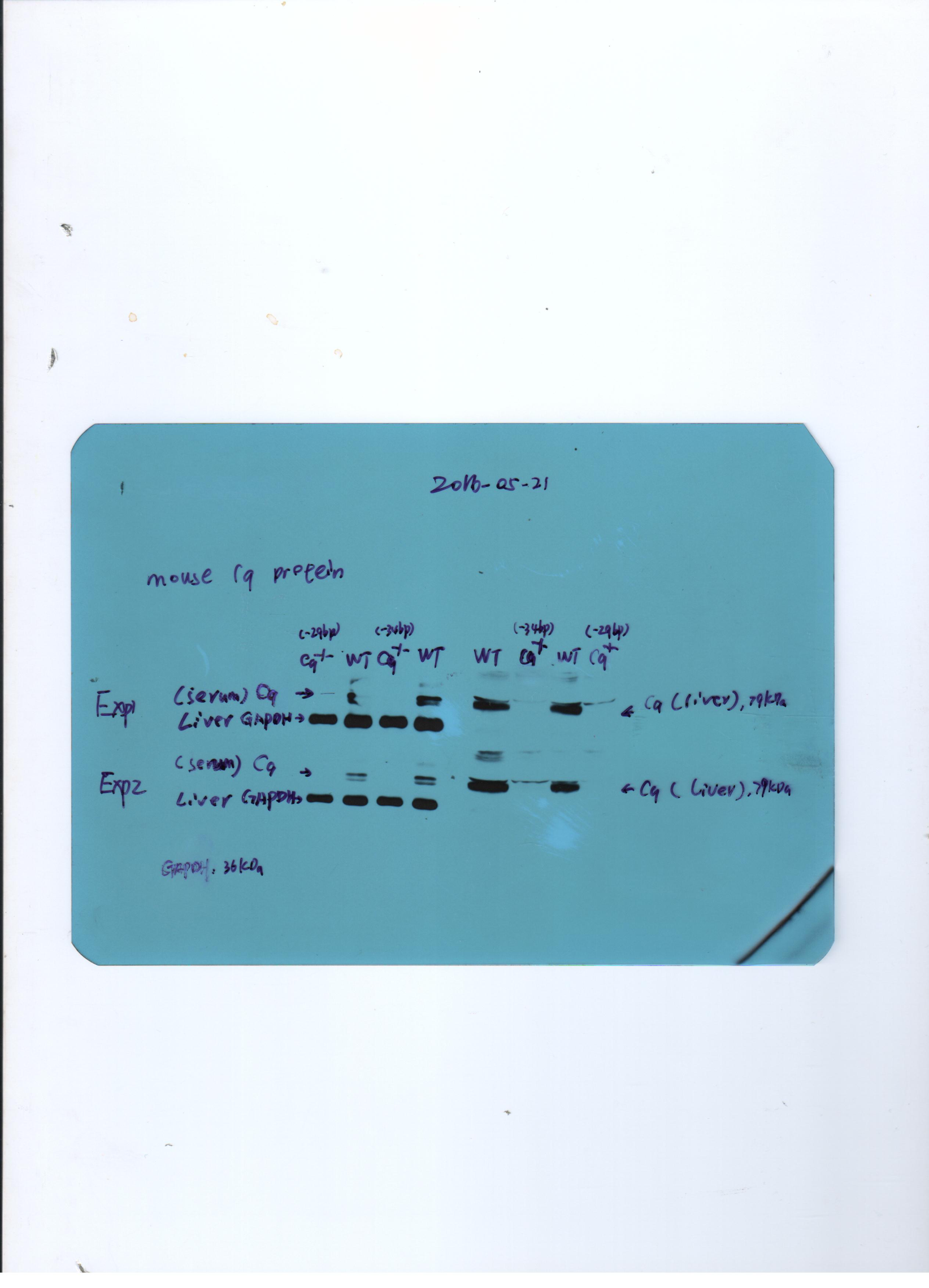
**

**Supplementary Figure 3:** Western blotting detection of C9 protein in liver tissue and serum from -34 bp and -29 bp founder. Protein samples from liver tissue or serum ware separated on 10% SDS-PAGE gel and were then subjected to western blotting with either rabbit anti - C9 poly – clonal antibody (1:200 dilution) or rabbit anti-GAPDH poly – clonal antibody (1:1,000 dilution).. Both -34 bp and -29 bp founder showed a lack of C9 protein in either liver tissue or serum. The blotting here showed two independent experiments.


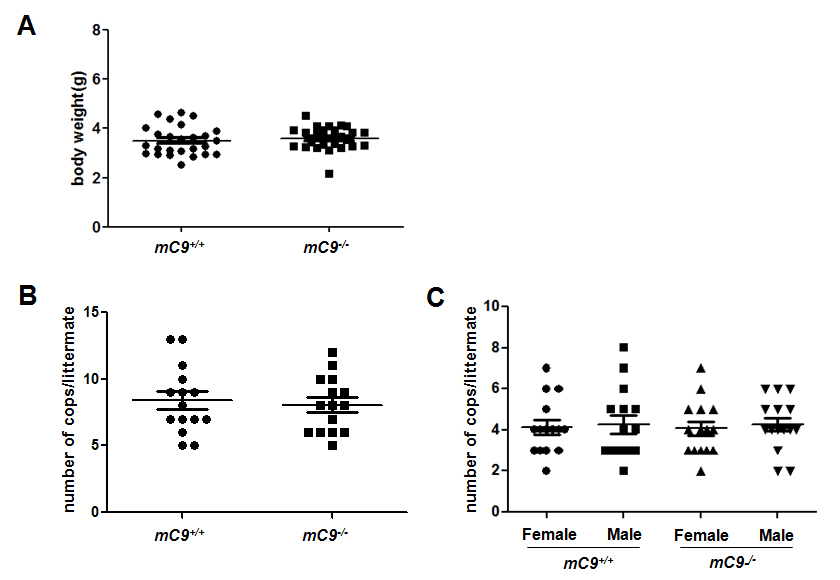


**Supplementary Figure 4:** There was no difference in body weight calculated at day 7 after birth (n=27 for each group) of new cops (**A**), fertility (15 littermates from each group) (**B**), gender (**C**), general activity and daily food intake among *mC9^+/+^* and *mC9^-/-^*.

**
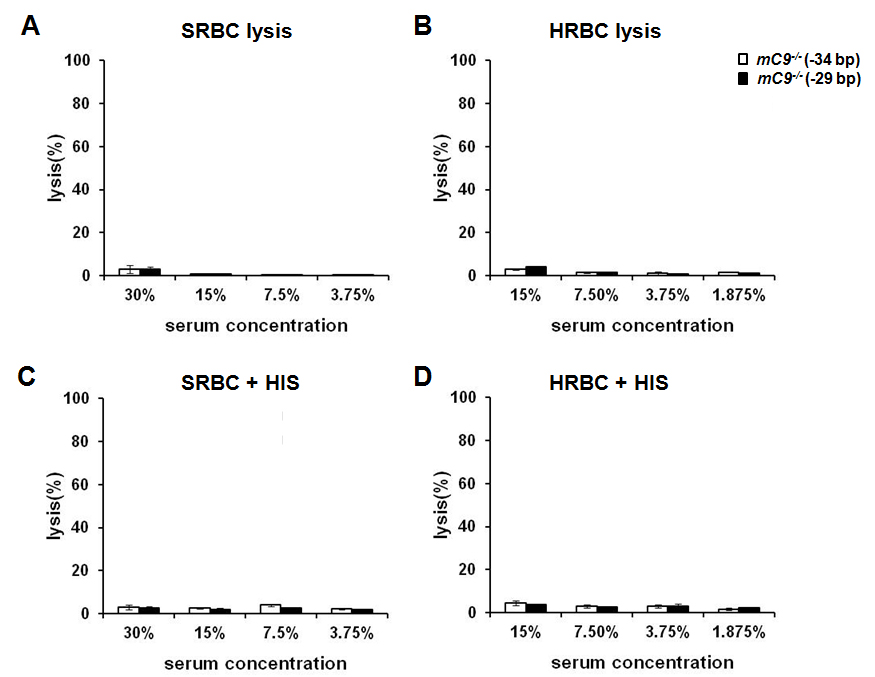
**

**Supplementary Figure 5:** Hemolysis with serum for -29 bp and -34 bp founder. Ability of -29 bp(n=5) or -34 bp (n=5) *mC9^-/-^* founders derived sera as a source of complement to lyse SRBCs (**A**) or HRBCs (**B**) during anti-RBC antibody mediated hemolytic assay was evaluated. There was no significant difference between -29 bp or -34 bp *mC9^-/-^* founders at any detected serum concentration. Heated inactivated serum (HIS) from either -29 bp (n=5) or -34 bp (n=5) *mC9^-/-^* founders did not mediate any hemolytic effect on either SRBCs (**C**) or HRBCs (**D**).

**
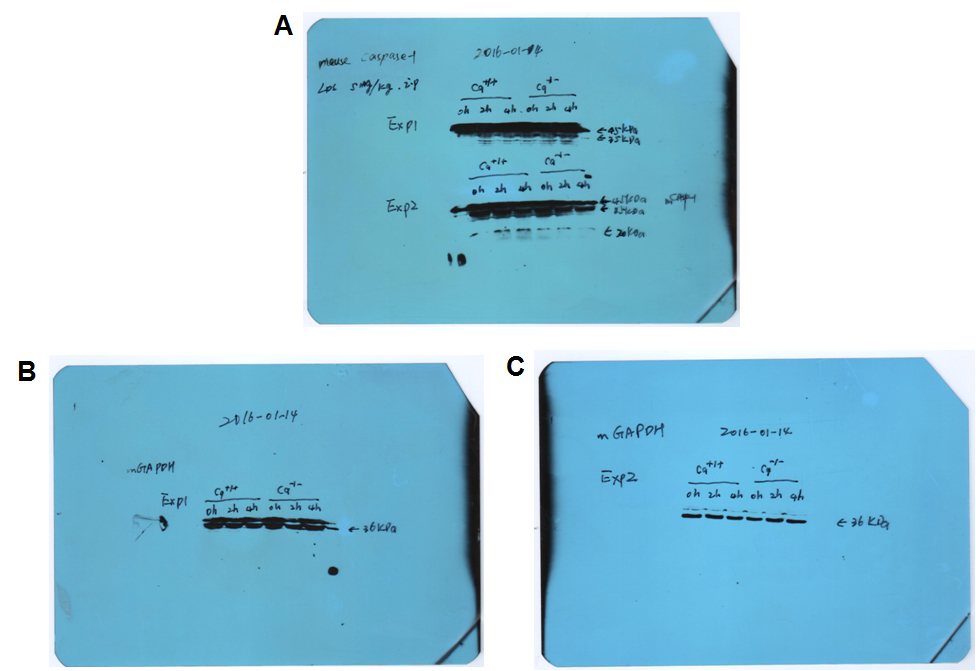
**

**Supplementary Figure 6:** Detection of caspase-1 activation in slpenocytes through western blotting. A total of 100 μg total protein per sample was separated on 15% SDS – PAGE gel and was then subjected to western blotting with either rabbit anti-caspase-1 poly – clonal antibody (1:1,000 dilution) (**A**)or rabbit anti-GAPDH poly – clonal antibody (1:1,000 dilution) (**B, C**). Compared to *mC9^+/+^* splenocytes, activation of caspase-1 decreased significantly in *mC9^-/-^* mice derived splenocytes. The result here showed two independent experiments.

**Supplementary Table**


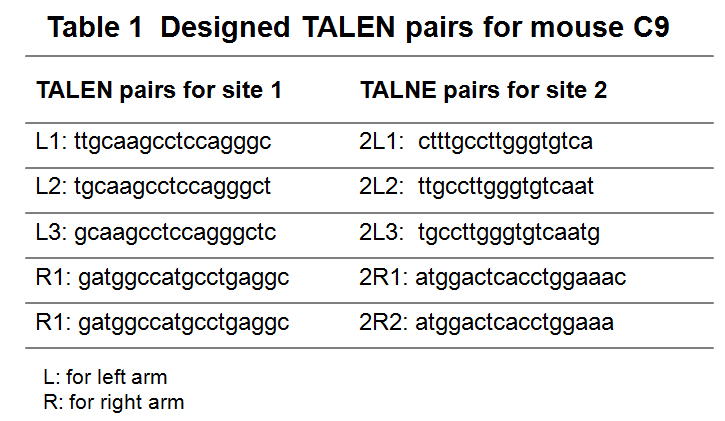


**Supplementary Table 1:** According to the *C9* gene of mouse in this project, we designed TALEN pairs in the form of 2×3 (one right arm combine one left arm) TALEN combinations for two different sites. Finally, 12 pairs of TALEN plasmid were obtained through restriction enzyme identification and sequencing.
